# Idiopathic mesenteric phlebosclerosis initially misdiagnosed as bowel obstruction: a case report

**DOI:** 10.3389/fmed.2026.1830460

**Published:** 2026-04-30

**Authors:** Binlong Zhang, Xiaomin Yang, Haocheng Zhao

**Affiliations:** 1 Department of Gastroenterology, Yueqing People’s Hospital, Yueqing, Zhejiang, China; 2 Department of Hematology, Yueqing People’s Hospital, Yueqing, Zhejiang, China

**Keywords:** bowel obstruction, CT imaging, diagnostic challenges, gardenia-containing herbal medicine, idiopathic mesenteric phlebosclerosis

## Abstract

Idiopathic Mesenteric Phlebosclerosis is a rare and misdiagnosed gastrointestinal condition marked by mesenteric venous sclerosis and calcification, which can lead to chronic mesenteric ischemia and colonic irregularities. This study reports a 68-year-old male with recurrent abdominal bloating and constipation, initially misdiagnosed as intestinal obstruction. The medical history of the patient included prolonged use of Gardenia Jinhua Wan, a herbal preparation containing Gardenia. Imaging studies, including CT scans, revealed calcified mesenteric veins and colonic wall thickening, while colonoscopy indicated deep blue mucosa with ulcerations and stricture, which are the characteristic features of IMP. Histopathological analysis validated chronic inflammation and submucosal fibrosis, and Masson trichrome-positive staining ruled out amyloidosis (Congo red-negative). The IMP diagnosis was confirmed after the discontinuation of the herbal remedy, while mesalazine and aspirin significantly alleviated symptoms. This case highlights the importance of considering IMP as the differential diagnosis of chronic gastrointestinal problems, particularly when associated with the use of herbal medicine. These findings suggest that early identification and imaging results, including venous calcifications and endoscopic mucosal discolouration, are crucial for preventing misinterpretation and unwarranted interventions.

## 1 Introduction

Idiopathic Mesenteric Phlebosclerosis (IMP) is a rare gastrointestinal disorder characterized by progressive sclerosis and mesenteric vein calcification, which leads to chronic mesenteric ischemia and associated colonic abnormalities (1). Furthermore, IMP has been observed to have nonspecific symptoms such as chronic abdominal pain, bloating, and altered bowel habits, which overlap with other common conditions like inflammatory bowel disease (IBD) and ischemic colitis (1–3). The characteristic findings of IMP have been studied *via* diagnostic imaging, such as computed tomography (CT) and colonoscopy, and include mesenteric vein calcifications and bowel wall thickening. Moreover, specific mucosal discoloration and ulcerations can be observed by endoscopic examination (4–7). However, due to the rarity of IMP, it is often underdiagnosed or misdiagnosed, presenting difficulties for physicians in making an appropriate diagnosis (2, 3).

Recently, several studies have associated IMP with the prolonged use of herbal medicines, specifically those containing Gardenia, such as Gardenia Jinhua Wan (8). This association is particularly important in individuals who have chronic gastrointestinal symptoms that persist even after conventional treatment (2). This study reports a 68-year-old male patient who was first diagnosed with intestinal blockage and treated conservatively, without additional investigations to ascertain the underlying reason. However, after 1 year of recurrent symptoms, IMP diagnosis was confirmed by comprehensive imaging and colonoscopy examination. It was determined that the patient had been using Gardenia Jinhua Wan for a prolonged duration, which might have promoted the disease onset (8).

This case illustrates the diagnostic difficulties presented by IMP, especially during the initial stages when symptoms resemble other more prevalent gastrointestinal disorders, including bowel obstruction (2, 3). Furthermore, it also highlights the significance of evaluating the impact of prolonged herbal medicine use on IMP onset (2, 8). Moreover, the characteristic IMP findings on CT imaging, colonoscopy, and histopathology underscore the need for a comprehensive and precise diagnostic strategy in rare diseases (4–7). The results of this report will raise awareness of IMP and its potential association with herbal treatments, providing significant insight into its diagnosis and management (2, 8).

## 2 Case presentation

A 68-year-old male was administered with a year of recurrent abdominal bloating and constipation. His symptoms included upper abdominal discomfort and difficulty passing stools, which were briefly alleviated by over-the-counter drugs. However, in January 2024, he was hospitalized due to the progressive worsening of these symptoms. During this admission, his abdominal CT revealed significant colon and rectum wall thickening, with multiple calcified mesenteric veins and alterations in pericolic exudate. Based on these findings, he was diagnosed with bowel obstruction and treated conservatively with fasting, gastrointestinal decompression, and fluid support. Despite symptomatic improvement, the underlying etiology was not further investigated, resulting in a missed diagnosis of IMP.

**Figure 1:**
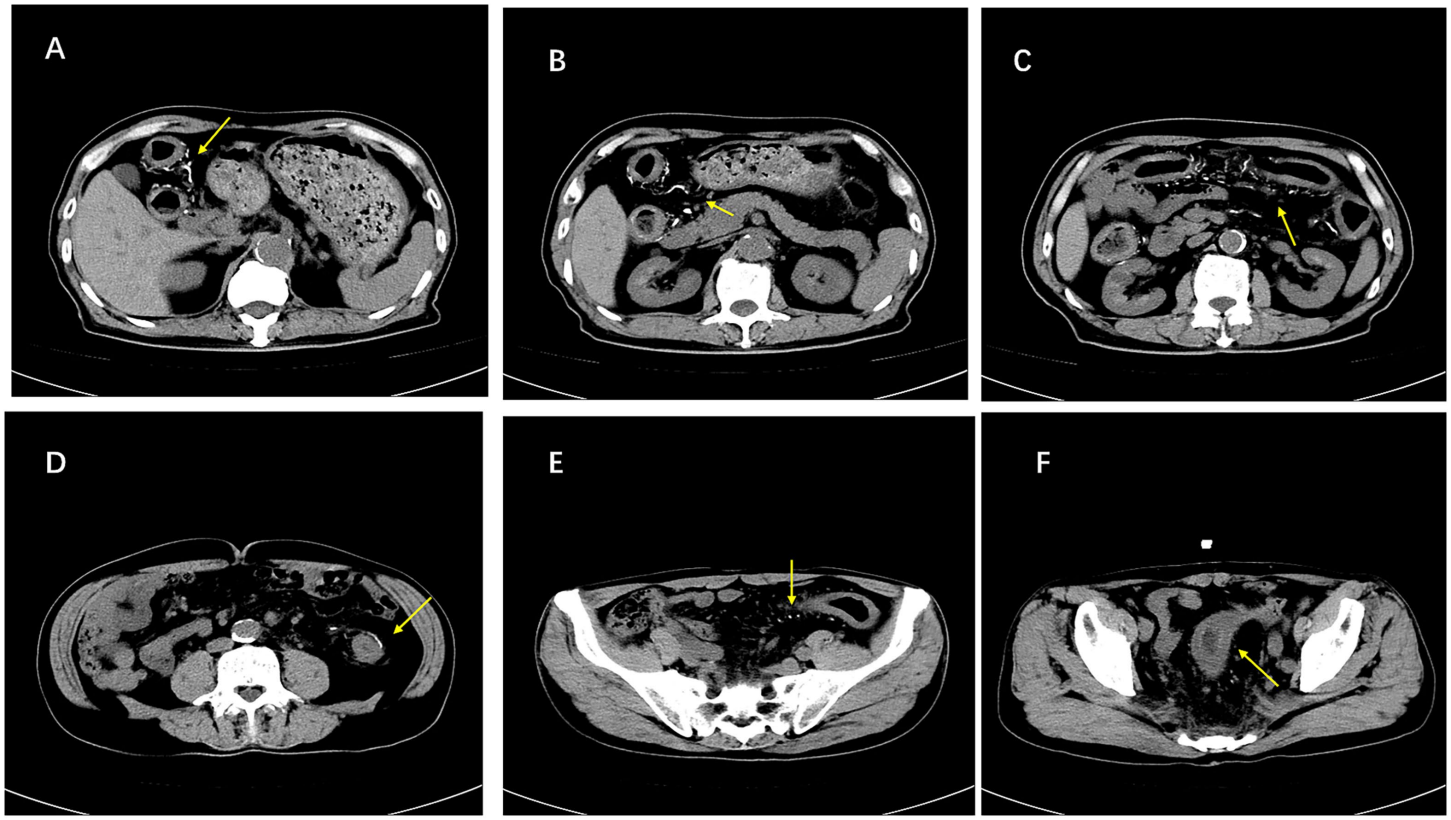
Abdominal CT images of idiopathic mesenteric phlebosclerosis (IMP). Non-contrast abdominal CT findings in IMP. **(A–D)** Axial images demonstrate circumferential thickening of the colonic wall with adjacent pericolic fat stranding, along with many thread-like hyperdense calcifications distributed along the pericolic and mesenteric veins (arrows). **(E,F)** Axial pelvic images show edematous thickening of the rectal wall with adjacent linear mesenteric/perirectal venous calcifications (arrows), consistent with IMP-related chronic ischemic change.

**Figure 2:**
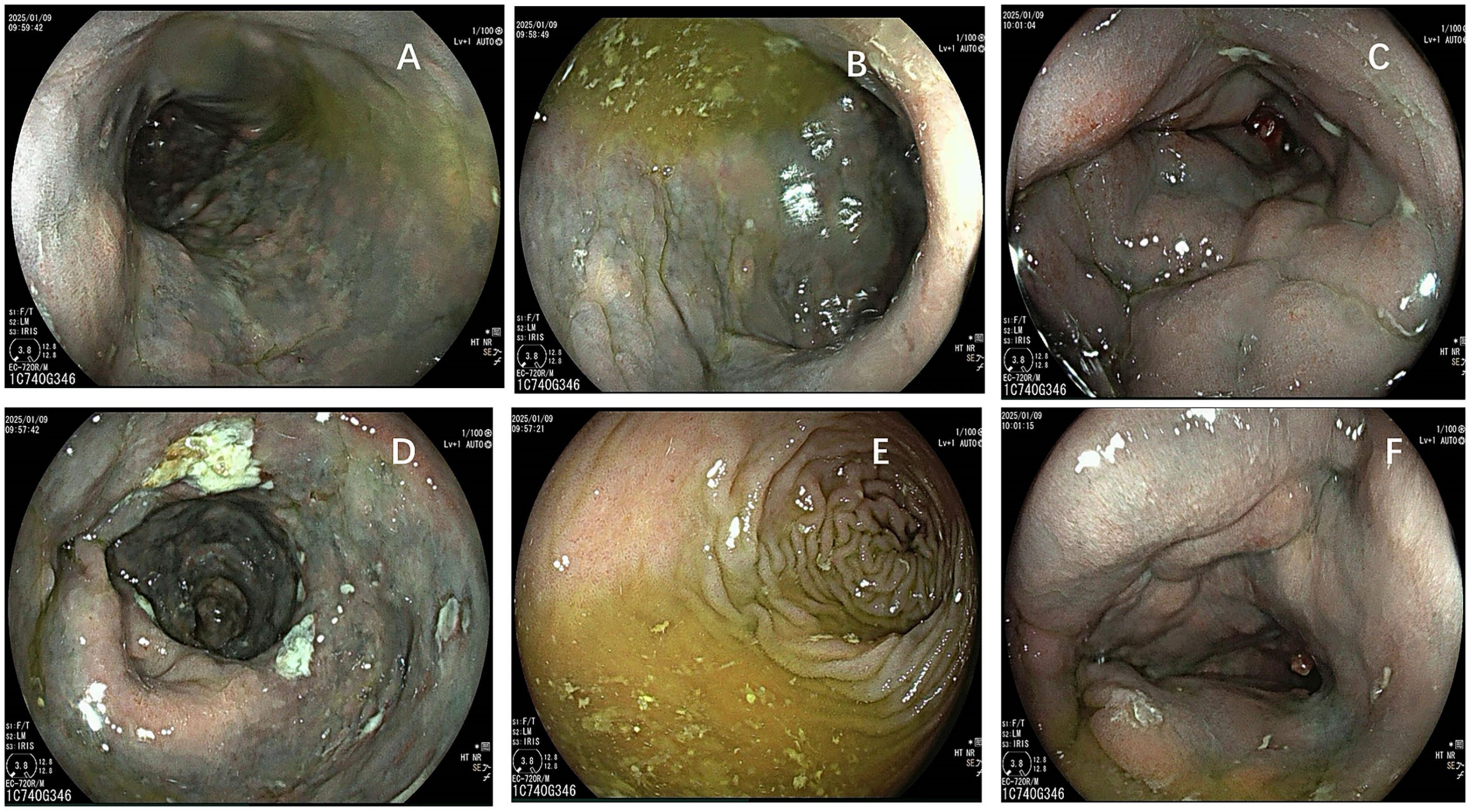
Colonoscopic findings in idiopathic mesenteric phlebosclerosis (IMP). **(A–D)** Colonic segments indicate diffuse dark blue to grayish-blue mucosal staining, significant mucosal edema, and luminal constriction, along with irregular mucosal erosions and shallow ulcerations with adherent exudate, indicative of chronic ischemic alterations. **(E)** Affected colon demonstrating rigid, thickened folds with an ischemic-appearing, discolored mucosa. **(F)** Additional view of the colon showing persistent mucosal swelling, reduced distensibility, and bluish discoloration, characteristic of IMP.

**Figure 3:**
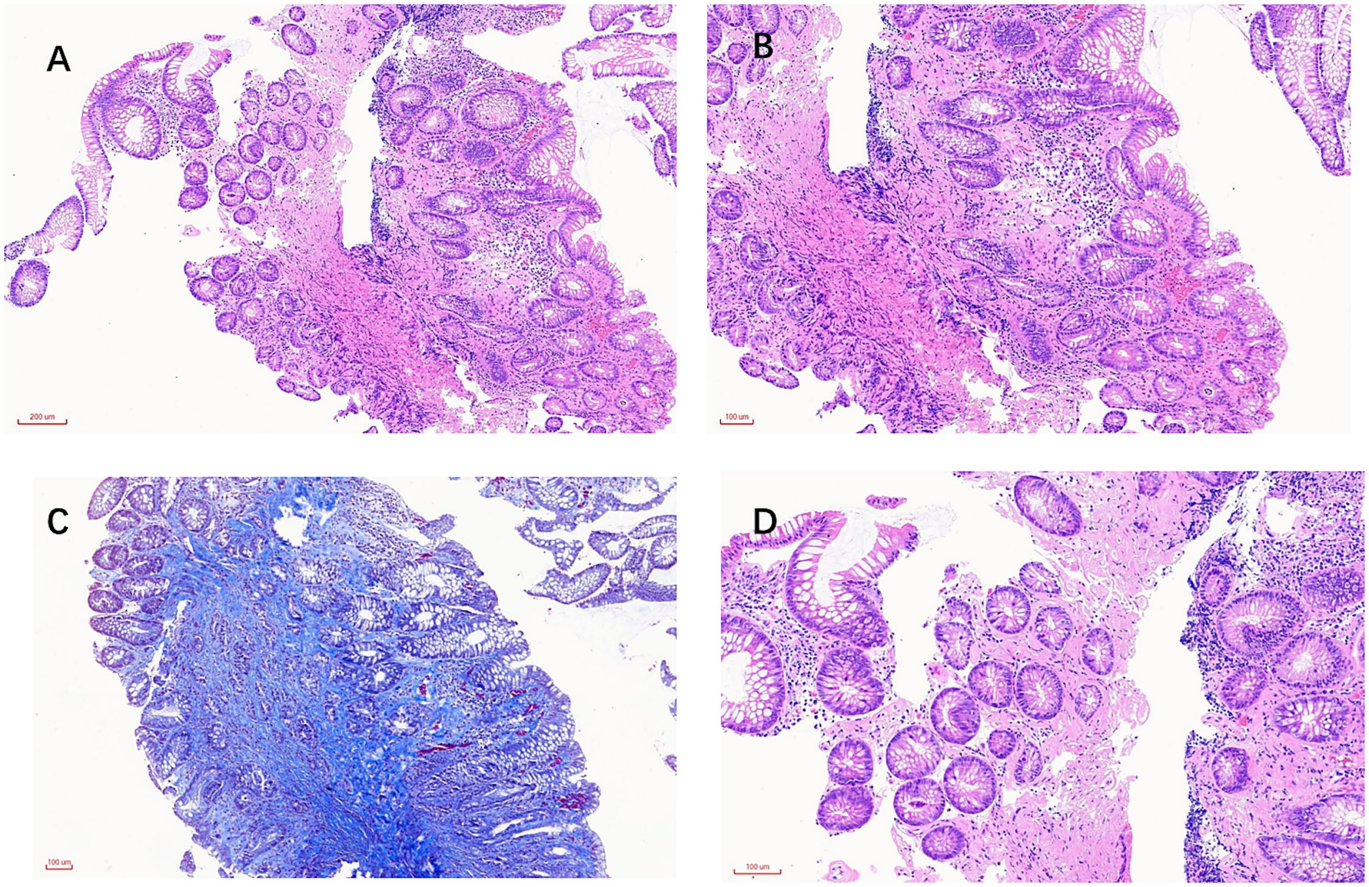
Histopathological features of the colonic mucosa in idiopathic mesenteric phlebosclerosis (IMP). **(A,B)** H&E-stained sections indicate mild crypt architectural alteration with a few mixed inflammatory cell infiltrations within the lamina propria, as well as stromal expansion consistent with early fibrous proliferation. **(C)** Masson’s trichrome stain shows elevated collagen deposition/fibrosis within the mucosa and lamina propria (trichrome-positive blue staining). **(D)** Higher-power H&E view highlights lamina propria inflammatory infiltrates with fibroblastic stromal proliferation, compatible with inflammatory granulation-type change. Congo red staining was negative. Because this case was based on limited endoscopic biopsy tissue rather than a surgical resection specimen, a typical sclerosed vein was not directly identified in the sampled tissue.

After 1 year, the patient returned with similar symptoms. He was experiencing episodes of diarrhea, characterized by yellow watery stools, after using laxatives. The physical examination in the gastroenterology department revealed mild bloating but no tenderness. Furthermore, laboratory tests indicated anemia (hemoglobin 98 g/L) and increased fecal calprotectin content. However, abdominal CT (Figure 1) and colonoscopy (Figure 2) findings were consistent with IMP, including mesenteric vein calcification and circumferential colonic wall thickening (Table 1). This diagnosis was confirmed by histopathology analysis (Figure 3), which indicated mild crypt changes, fibrosis, and inflammatory infiltration in the colonic mucosa. Patient’s history revealed a prolonged use of Gardenia Jinhua Wan, a Chinese herbal remedy, as a primary cause of IMP.

**Table 1:** Computed tomography and colonoscopic findings in the present case.

Feature	CT findings	Colonoscopy findings
Mesenteric vein calcification	Thread-like/linear calcifications along mesenteric vein branches (arrows)	Not applicable
Colonic wall thickening	Circumferential thickening of the ascending colon and cecum (arrows)	Not applicable
Colon	Predominant impairment of the right colon (ascending colon, cecum)	The right colonic half is predominantly affected (arrows)
Mucosal discoloration	Not applicable	Deep blue/grayish-blue discoloration in the right colon (arrows)
Mucosal erosions/ulcerations	Not applicable	Multiple shallow irregular ulcerations and erosions (arrows)
Luminal narrowing	Not applicable	Luminal narrowing, associated with ischemic changes (arrows)

After discontinuing Gardenia Jinhua Wan, the patient was treated with mesalazine and aspirin, which substantially improved his symptoms, as well as reduced abdominal bloating and constipation. His clinical condition became stable, and he was constantly monitored. This case highlights the diagnostic challenges of IMP, particularly in its initial phases when symptoms resemble other more prevalent gastrointestinal disorders, such as bowel obstruction, and highlights the potential association of prolonged herbal medicine use with IMP onset.

## 3 Discussion

Idiopathic Mesenteric Phlebosclerosis is an uncommon gastrointestinal condition characterized by persistent mesenteric ischemia resulting from the gradual sclerosis and calcification of mesenteric veins (1). This disorder generally manifests with nonspecific symptoms, including stomach discomfort, bloating, and constipation, which may be readily mistaken for other more prevalent gastrointestinal conditions, such as IBD or ischemic colitis (1–3). Its diagnosis primarily relies on distinctive imaging characteristics such as mesenteric vein calcification and colonic wall thickening, observable via CT scans (4, 5). Furthermore, endoscopic evaluation often indicates characteristic mucosal discoloring, including deep blue or grayish-blue mucosa, which is a hallmark of this condition (6, 7). Therefore, for the accurate IMP diagnosis, these imaging and endoscopic features, as well as the patient’s clinical presentation, are essential (1).

Several studies have associated IMP with long-term use of Gardenia-containing traditional Chinese herbal products. In practice, the relevant exposure is usually through herbal medicinal products rather than common food products, and it may occur either as a stand-alone crude herb (*Gardenia jasminoides* fruit, “Zhi Zi”) or, more commonly, as part of multi-herb formulations; in the present case, the patient had prolonged exposure to Gardenia Jinhua Wan, a compound herbal preparation containing Gardenia (8). Here, the patient had been using Gardenia Jinhua Wan for a prolonged period, which might have led to IMP onset (8). Gardenia comprises geniposide, a compound that is metabolized to genipin, which may induce venous fibrosis and sclerosis in the mesenteric veins (8). Although the specific mechanism continues be studied, genipin has been found to induce fibrotic alterations in the mesenteric veins, thus promoting IMP development (8). The correlation between herbal medicine use and IMP highlights the significance of comprehensively evaluating a patient’s history, including the use of over-the-counter treatments, in cases of unexplained gastrointestinal symptoms (2, 8, 9).

This report also illustrates the diagnostic difficulties of IMP, particularly during its initial phases, where clinical presentations may coincide with more prevalent disorders such as intestinal obstruction (detailed in Table 2) (2, 3). The patient was initially diagnosed with bowel blockage and managed conservatively; however, the underlying reason was not identified until a year later, when an additional investigation identified the true etiology. The CT findings and colonoscopy results revealed calcified mesenteric veins and thicker colonic walls, which were crucial in confirming the diagnosis (4–7). Because only limited endoscopic biopsy tissue was available in this case, a typical sclerosed vein was not directly demonstrated histologically, and the diagnosis relied primarily on the characteristic computed tomography findings together with the colonoscopic and biopsy features. The patient’s symptomatic improvement after discontinuing the herbal medicine and treatment with mesalazine and aspirin suggests that Gardenia-based treatments might be the possible cause of IMP development. Despite IMP being a rare illness with no conventional treatment protocols, this case illustrates that discontinuation of the causative herbal drug, as well as the symptomatic therapy, can lead to significant clinical improvement (2, 8, 10).

**Table 2:** Differential diagnostic considerations based on computed tomography and colonoscopic findings in the present case.

Conditions	CT findings	Colonoscopy findings	Patient’s key differentiation features
IMP	Thread-like/linear calcifications along mesenteric vein branches; ascending colon and cecum’s circumferential wall thickening; predominant right colon involvement	Cecum to transverse colon deep blue/slate-gray mucosal discoloration; loss of normal haustral pattern; luminal narrowing; multiple shallow irregular ulcerations	Characteristic venous calcifications on CT; deep blue mucosa on colonoscopy; right colon predominance
Bowel obstruction	Possible colonic wall thickening; venous calcification (not a defining feature).	Not available	Explains bloating and constipation, but not mesenteric venous calcifications or diffuse blue mucosal discoloration
Ischemic colitis	Colonic wall thickening; absent venous calcification	Erythema; edema; ulceration; blue/slate-gray discoloration (uncommon)	Absence of characteristic thread-like mesenteric venous calcifications; mostly segmental distribution instead of predominantly right-sided
IBD	Possible wall thickening; venous calcification (uncommon)	Erosions and ulcerations; erythematous mucosal discoloration (not blue)	Absence of venous calcification and characteristic blue-gray mucosa
Colonic amyloidosis	Not available	Mucosal friability or possible ulceration	Negative Congo red staining, excluding amyloid deposition

## 4 Conclusion

IMP is an uncommon and frequently misdiagnosed disorder marked by nonspecific gastrointestinal symptoms, including abdominal bloating and constipation. The diagnosis is mostly based on distinct imaging irregularities, such as calcified mesenteric veins and thicker colonic walls, as well as the characteristic deep blue mucosa observed during colonoscopy. This case study highlights the difficulties of diagnosing IMP, especially when initial symptoms resemble other, more prevalent conditions such as intestinal obstruction, as observed in this patient. The patient’s prolonged use of Gardenia Jinhua Wan was found to be the primary cause, as Gardenia-derived medications have been associated with IMP due to the effects of geniposide and its metabolite, genipin, which may facilitate venous sclerosis. The misdiagnosis of intestinal obstruction highlights the necessity of including rare illnesses such as IMP in the differential diagnosis, especially in patients with a history of herbal medication usage. Furthermore, symptomatic improvement was achieved after the herbal medicine was discontinued and the patient was treated with mesalazine and aspirin, indicating a possible drug-related etiology. These findings suggest that although the treatment approach for IMP remains non-standardized, early identification, discontinuation of the causative drug, and conservative therapy can substantially mitigate symptoms. Therefore, IMP should be included in the differential diagnosis for patients exhibiting chronic gastrointestinal symptoms, especially those with a history of herbal remedy use, as timely identification can prevent unnecessary interventions.

## Data Availability

The raw data supporting the conclusions of this article will be made available by the authors, without undue reservation.
